# Accumulated source imaging of brain activity with both low and high-frequency neuromagnetic signals

**DOI:** 10.3389/fninf.2014.00057

**Published:** 2014-05-21

**Authors:** Jing Xiang, Qian Luo, Rupesh Kotecha, Abraham Korman, Fawen Zhang, Huan Luo, Hisako Fujiwara, Nat Hemasilpin, Douglas F. Rose

**Affiliations:** ^1^Division of Neurology, MEG Center, Cincinnati Children's Hospital Medical CenterCincinnati, OH, USA; ^2^Department of Neurosurgery, Saint Louis UniversitySt. Louis, MO, USA; ^3^Cleveland Clinic Foundation, Department of Radiation OncologyCleveland, OH, USA; ^4^Department of Communication Sciences and Disorders, University of CincinnatiCincinnati, OH, USA; ^5^State Key Laboratory of Brain and Cognitive Science, Institute of Biophysics, Chinese Academy of SciencesBeijing, China

**Keywords:** magnetoencephalography, brain, multi-frequency, high-frequency oscillations, magnetic source imaging

## Abstract

Recent studies have revealed the importance of high-frequency brain signals (>70 Hz). One challenge of high-frequency signal analysis is that the size of time-frequency representation of high-frequency brain signals could be larger than 1 terabytes (TB), which is beyond the upper limits of a typical computer workstation's memory (<196 GB). The aim of the present study is to develop a new method to provide greater sensitivity in detecting high-frequency magnetoencephalography (MEG) signals in a single automated and versatile interface, rather than the more traditional, time-intensive visual inspection methods, which may take up to several days. To address the aim, we developed a new method, accumulated source imaging, defined as the volumetric summation of source activity over a period of time. This method analyzes signals in both low- (1~70 Hz) and high-frequency (70~200 Hz) ranges at source levels. To extract meaningful information from MEG signals at sensor space, the signals were decomposed to channel-cross-channel matrix (CxC) representing the spatiotemporal patterns of every possible sensor-pair. A new algorithm was developed and tested by calculating the optimal CxC and source location-orientation weights for volumetric source imaging, thereby minimizing multi-source interference and reducing computational cost. The new method was implemented in C/C++ and tested with MEG data recorded from clinical epilepsy patients. The results of experimental data demonstrated that accumulated source imaging could effectively summarize and visualize MEG recordings within 12.7 h by using approximately 10 GB of computer memory. In contrast to the conventional method of visually identifying multi-frequency epileptic activities that traditionally took 2–3 days and used 1–2 TB storage, the new approach can quantify epileptic abnormalities in both low- and high-frequency ranges at source levels, using much less time and computer memory.

## Introduction

Recent studies have revealed the significance of high-frequency brain signals – such as high-frequency oscillations (HFOs, 90–200 Hz), ripples (80–250 Hz) and fast ripples (250–500 Hz) relative to the conventional lower frequency brain signals (<70 Hz) (Pulvermuller et al., 1997; Guggisberg et al., 2007; Gotman, 2010; Worrell et al., 2012). One of the important motivations behind the study of high-frequency brain signals is their potential clinical applications. HFOs may be important biomarkers of epileptogenicity, a revolutionary finding revealed in recent years (Xiang et al., 2004, 2009a, 2010). Clinical data have revealed that removal of HFO-generating areas lead to improved surgical outcomes (Haegelen et al., 2013). In addition, by using HFOs, it is possible to substantially reduce the extent of cortical resections in epilepsy surgery procedures without compromising seizure control (Weiss et al., 2013). Furthermore, HFOs also play a very important role in many brain disorders (Uhlhaas et al., 2011). For example, schizophrenia is associated with abnormal amplitude and synchrony of high frequency activities (Uhlhaas and Singer, 2013). Of note, the study of high-frequency brain signals may shed light on some of the fundamental mechanisms of neuronal functions and brain disorders.

Numerous challenges exist in the study of high-frequency brain signals with magnetoencephalography (MEG) and electroencephalography (EEG) (Xiang et al., 2004, 2010, 2013; Dalal et al., 2008; Papadelis et al., 2009; Chen et al., 2010; Gotman, 2010; Gummadavelli et al., 2013). First, the size of high sampling rate data can be over 12 terabytes (TB) (Blanco et al., 2011). The size of high sampling rate data can cause a substantial amount of data, posing a challenge for data transfer, storage, archiving, sharing and analysis (Van Essen et al., 2012; Worrell et al., 2012; Zafeiriou and Vargiami, 2012; Zijlmans et al., 2012b). Given the massive amounts of high-sampling rate MEG/EEG data that are collected from patients and research subjects, it is impractical to rely on a visual review of HFOs (Haegelen et al., 2013; Tort et al., 2013; Xiang et al., 2013). Second, in clinical practice, MEG/EEG data are typically analyzed with other neuroimaging data such as invasive recordings, magnetic resonance imaging (MRI) and functional MRI (fMRI). The considerable volume of multi-modal neuroimaging data produced across different communities has posed a daunting challenge to the traditional methods of data sharing, data archiving, data processing, and data interpreting (Van Essen et al., 2012; Worrell et al., 2012; Zafeiriou and Vargiami, 2012; Zijlmans et al., 2012b). Though the multi-modal data enhance our collective understanding of the structure and function of the brain, it is a challenge to handle these varied and heterogeneous datasets. Even with modern computational innovations, there remain technical challenges in data transfer, storage, and analysis of large data sets of more than 12 TB (Brinkmann et al., 2009; Le Van Quyen et al., 2010). Third, the best way to clinically utilize analysis from high-frequency brain signals remains a challenge. While it has been demonstrated that the brain generates signals in wide frequency ranges, there are currently no established criteria for distinguishing physiologic high-frequency signals from pathologic neuromagnetic signals (Worrell et al., 2012; Zijlmans et al., 2012b; Haegelen et al., 2013; Matsumoto et al., 2013; Pail et al., 2013; Srejic et al., 2013; Tort et al., 2013). Although multiple studies with invasive recordings have shown the feasibility and potential clinical importance of detecting HFOs (Jirsch et al., 2006; Engel et al., 2009; Jacobs et al., 2009, 2012; Levesque et al., 2011; Andrade-Valenca et al., 2012; Dumpelmann et al., 2012; Zijlmans et al., 2012b), there is no noninvasive method which can be used for clinical purposes. One remaining important clinical question is whether a noninvasive method can extract and visualize meaningful HFOs from the brain for research and clinical purposes.

This study aimed to resolve the aforementioned challenges associated with large scale high-frequency signal processing by developing novel analysis methodologies and workflows for the MEG data. Since the computer memory limits for a 32 bit and 64 bit operating system are 4 GB and 192 GB (Windows 7, respectively) (http://msdn.microsoft.com/en-us/library/windows/desktop/aa366778(v=vs.85).aspx) and the size of high-frequency brain signals are usually larger than 12 TB (Blanco et al., 2011), one methodological question this study would like to address is whether new algorithms could minimize the use of computer memory and storage. To solve the challenges of analyzing more than 12 TB of both high and low frequency MEG data, we mathematically and experimentally developed a systematic approach to extract meaningful frequency specific and spatiotemporal information from MEG data. Accumulated spectrograms, a technique which maximizes the signal power of the frequency of interest while simultaneously minimizing other frequency contents, provides a novel method of quantifying and visualizing the frequency signatures of brain activity in both low- and high-frequency ranges. Accumulated source imaging, which volumetrically reconstructs source activity in multiple frequency ranges, provides source images for clinicians to analyze epileptic activity at source levels. The central hypothesis of our research is that neuromagnetic brain signals in both low and high frequency ranges could be localized and visualized with accumulated source imaging. The new algorithm calculated optimal channel-cross-channel (CxC) matrices and source location-orientation weights for volumetric source imaging, minimizing multi-source interference and reducing computational cost. To demonstrate the advancements of the new methods in research and clinical settings, MEG data from subjects were obtained, analyzed, and demonstrated in 2D and 3D environments.

## Materials and methods

### Detection of low- and high-frequency MEG signals at sensor levels

**Figure 1:**
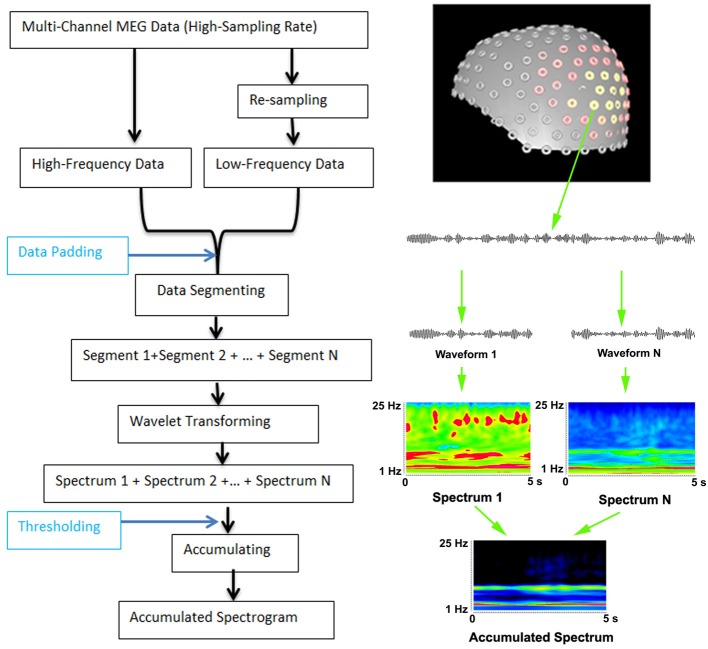
**Workflow for computing accumulated spectrogram (left) and the basic principle of computing accumulated spectrogram (right)**. Since the analysis of high-frequency MEG signals requires high-sampling rate MEG data, MEG data are digitized in a high-frequency range. To improve the performance and optimize the use of computer memory for analyzing both low- and high-frequency MEG signals, the new method can re-sample MEG data dynamically according to the analysis frequency ranges. If the data points of the recorded data are smaller than the minimum data point of wavelet transform in frequency range, the “Data Padding” function can pad some data points so as to meet the requirements of wavelet transform. The “Thresholding” indicates that a spectral value can be rejected or accepted by the accumulated spectrogram according to a threshold value. MEG data recorded are waveforms, which are divided to segments (e.g., “Waveform 1,” “Waveform N”) to minimize the use of memory for wavelet transform (“Wavelet transform”). In the new method, wavelet transform transfers each segment of waveform data to a spectrum (e.g., “Spectrum 1,” “Spectrum N”). Of note, “N” indicates the total number of segments or spectra, which can be theoretically infinitely large. The “+” indicates the process of accumulation, which add all spectra together to produce an accumulated spectrum (“Accumulated Spectrum”). The left view of the sensor distribution of our MEG system is shown on the top right.

Multi-channel MEG data had to be digitized at a high sampling rate because the sampling rate must be at least two times higher than the frequency edge of interest. For the analysis of low-frequency signals, MEG data could be resampled to minimize the use of memory and to improve the computational efficiency. Resampling was done by decimating signals to extract the low frequency data. A low-pass anti-aliasing filter was applied before resampling. The high and low frequency pass-bands depended on the sampling rate and the frequency ranges of interest. In this study, two pass-bands of 1–70 Hz and 70–200 Hz were used. To compute the accumulated spectrogram, filtered MEG data were then segmented into small data segments. The length of the data segments depended on the time window of wavelet-transformation. In this study, we used a 5 s time-window and 600 frequency bins. There was no overlap between segments. Of note, the total length of recorded MEG data did not always match exactly with all of the segments. To solve this problem, data padding (typically, adding zero to make up enough data points for computing) was applied. If there were more than enough data points, the program also allowed for discarding of “extra” data points. The time duration of these segments depended on several factors including the available computer memory, storage spaces and research purposes. Once the time-frequency representations were computed, they were accumulated into one spectrum by adding them together. The “threshold” was used during data accumulating. There were two threshold values: a minimum threshold value and a maximum threshold value. If a time-frequency value was smaller than the minimum threshold value (e.g., background activity) or larger than the maximum threshold value (e.g., artifacts), the value was discarded. Accumulated spectrum is different from an averaged spectrum because the process of accumulating has several parameters: (1) accumulating has two thresholds; and (2) the accumulated data do not have to be averaged. Since the analysis of high-frequency components required high-sampling data, the re-sampling function was critical for low-frequency spectral analysis, which also minimized the use of computer memory. The workflows of data analyses at sensor levels are illustrated in Figure 1.

Morlet continuous wavelet transform was used for transforming time-domain data to frequency-domain data (see Figure 1). The Morlet wavelet was used because brain activity is nonstationary and the wavelet is better suited for nonstationary data (Ghuman et al., 2011). Wavelet transform can be described by the following equation:

(1) $$G(t,\;f)=\frac{1}{\sqrt{2\pi f}}e^{\left(\frac{-t^{2}}{2\sigma^{2}}\right)}e^{i2\pi ft}$$

In the above formula, *t* indicates time, *f* indicates frequency, and σ represents the standard deviation of the Gaussian curve in the time domain. To ensure stability of the wavelet transform, σ is typically larger than $\frac{5}{2\pi f}$. Since the wavelet convolution brings Gaussian temporal blurring with a standard deviation of σ, the effective number of independent samples is $\frac{N-1}{\sqrt{2\pi {\left(f_{s}\sigma \right)}^{2}}}$. The *f*_*s*_ represents the sampling frequency of the data and *N* represents the number of data points.

Since brain activation in a given time-window might occur in different frequency ranges and different frequencies might have different corresponding amplitudes, we used a different sigma value for each frequency to capture the time-frequency changes. Consequently, wavelet Equation (1) can be represented with an alternate representation in Equation (2) as follows:

(2) $$G(t,\;f)=C_{\sigma }\pi^{-\frac{1}{4}}e^{-\frac{1}{2}t^{2}}(e^{i\sigma t}-\kappa_{\sigma })$$

In the formula, *t* indicates time and *f* indicates frequency. Each wavelet transform has its own sigma value. Sigma is the scaling parameter that affects the width of the window. The sigma values are derived from the mother function in wavelet transform by computing the number of small waves for a time-frequency analyses (Ghuman et al., 2011). Sigma values could also be experimentally determined. κ_σ_ represents the admissibility and *C*_σ_ represents a normalized constant. σ represents the standard deviation of the Gaussian curve in the time domain. If signals appeared in the given sensitive time (a small sigma value) and sensitive frequency (a large sigma value) ranges, they would be enhanced.

An accumulated spectrum was defined as the time-frequency summation of a long-time or continuous recording which had a time period at least two times longer than that of the time window of the spectrum. The equation of computing accumulated spectra is given by:

(3) $$Atf(s,\;f)=\sum_{t=1}^{T}\sum_{f=1}^{F}G(t,\;f)$$

In Equation (3), *Atf* represents an accumulated spectrum; *s* indicates the time slice of the spectrum; *f* indicates frequency bands (or bins) of MEG data; *T* indicates total time points of MEG data and *F* indicate the total frequency bands. We defined *s* ≥ 1 and *s* ≤ *T*/2. From computer program point of view, the use of computer memory and storage space by Equation (3) depends on the *s*. Even though *T* could be infinitively increasing, the requirements for computer memory and storage remain the same. Consequently, the approach automatically avoided possible “overflow” or “out of space” problems in a long-time or continuous recording for capturing epileptic activity.

An accumulated spectrogram was computed by sequentially transforming each of the segments of waveform data to time-frequency representations using Morlet wavelet algorithm Equation (2) and then accumulating all the spectra together Equation (3). In this procedure, the different spectrograms of individual time segments were mathematically summed together to a single new overall spectrogram. An accumulated spectrogram can reveal brain activity in a consistent frequency range at multiple time windows. It can be considered as a “collective result” for a long-time recording. Figure 1 demonstrates the basic principles of computing an accumulated spectrogram. An accumulated spectrogram could reveal brain activity in a consistent frequency range while minimizing noise at random frequency ranges (Figure 1). Therefore, it could be considered to be a “collective result” of spatial- and frequency locked signals in multiple epochs of MEG data. To identify the frequency profile of the entire brain for a recording, we developed an accumulated global spectrogram. An accumulated global spectrogram was an averaged spectrogram of all accumulated spectrograms from the entire MEG sensor array. The accumulated global spectrogram was the “spatial summation” of the entire MEG sensor array' accumulated spectrograms. Since each sensor was positioned in a distinct location around the brain if there was a subject, an accumulated global spectrogram should represent the magnetic field of the entire brain. The mathematical principles have been described in previous reports (Rau et al., 2002). The neuromagnetic activity at each sensor was visualized with contour maps, which showed small spectrograms at the position of each MEG sensor. The equation of computing global spectrogram is given by:

(4) $$G(s,\;f)=\frac{1}{M}\sum_{m=1}^{M}Aft(s,\;f)$$

In Equation (4), *G* represents the global spectrogram; *Atf* represents an accumulated spectrum of one MEG sensor data; *m* indicates MEG sensor index and *M* indicates the total number of MEG sensors; *s* indicates the time slice of the spectrum; *f* indicates frequency bands (or bins) of MEG data. Since each sensor was positioned in a distinct location around the head (Figure 1), the global spectrogram is considered to be a “spatial summation” for each epoch of data (Xiang et al., 2009a).

### Detection of low- and high-frequency MEG signals at source levels

**Figure 2:**
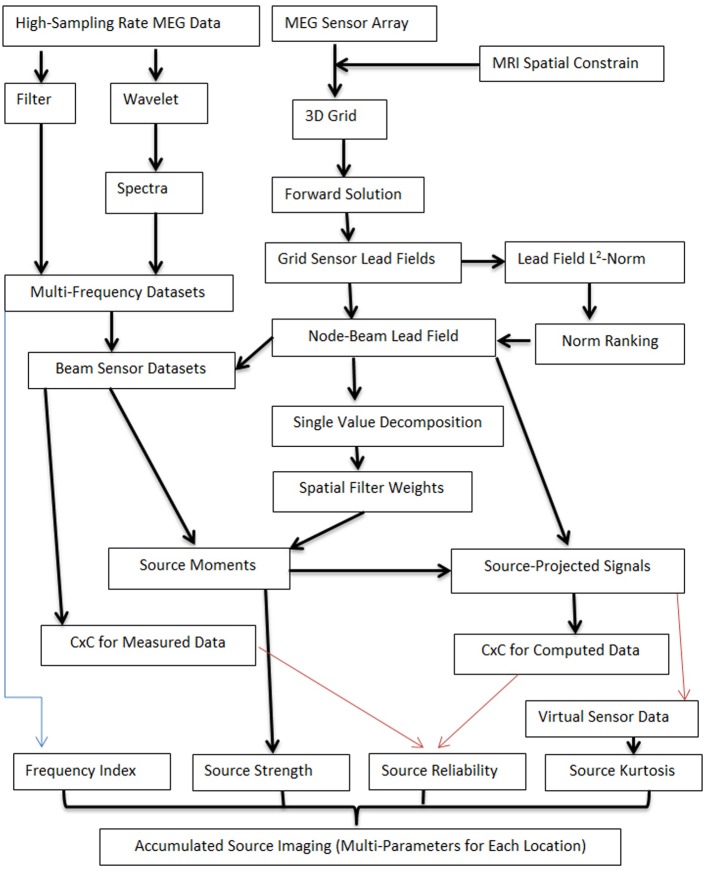
**Workflow for computing accumulated source images**. The workflow includes two main computing pipelines. One computing pipeline processes MEG data with filter or wavelet transforms so as to generate multi-frequency datasets. MEG signals in multi-frequency datasets are in a set of frequency ranges. Another computing pipeline works on several tasks, which included the creation of a three-dimensional source grid (3D grid), performing forward solution by calculating lead fields, ranking the norm for each source for all sensors, and performing SVD. The node-beam lead field is completed by selecting a group of sensors which have a larger lead field norm (or weights). Of note, each location in accumulated source imaging can have multiple parameters (e.g., “Frequency Index,” “Source Strength”). Some processes are optional (red lines) and additional parameters can also be added to the workflow.

To detect low- and high-frequency neuromagnetic signals at source levels, two computing pipelines were developed. One computing pipeline generated multi-frequency datasets by processing MEG data with filter or wavelet transforms. MEG signals in multi-frequency datasets were in a set of frequency ranges. Of note, the frequency ranges depended on the research tasks and can be predefined. Another computing pipeline performed four tasks: (1) creating a three-dimensional source grid (3D grid), where each grid node represents a possible source; (2) conducting forward solution by calculating lead fields for each source (node) for the entire grid; (3) computing the lead field norm (or magnitude) and ranking the norm for each source for all sensors; (4) producing the node-beam lead field, performing single value decomposition (SVD) and calculating spatial filter weights. The node-beam lead field, which represents a form of sub-space solution, was completed by selecting a group of sensors which had a larger lead field norm. According to our tests, the optimal number of sensors for a node-beam lead field was in a range of 3 to M/3; here M indicates the total number of sensors of a whole cortex MEG system. For example, in our study, the total number of MEG sensor was 275. Thus, the suitable number of sensors that could be used for node-beam lead field was 3–91 (275/3). Of course, all sensors could be used for source scan. A small number of sensors was used in node-beam lead field because high-frequency brain signals were typically very weak and appeared only in a focal group of sensors. The node-beam sensors were also used to generate beam sensor MEG datasets so that the sensors in forward solution matched with measured magnetic signals. The final step was to compute source moments and to generate source data. Additional components were optional (red lines, which will be discussed in following sections). The main workflow for localizing both low- and high-frequency MEG signals is shown in Figure 2.

Differing from the conventional volumetric source imaging or distributed source map, each grid node consisted of multiple data items including the strength and frequency of the source activity (Figure 2). Building on previous reports (Mosher and Leahy, 1998; Vrba and Robinson, 2001; De Gooijer-Van De Groep, 2013), the mathematic relationship between measured MEG data and source activity can be expressed as following equation:

(5) B=LQ+N

In Equation (4), *B* represents the MEG data; *L* represents the lead field, *Q* represents the source strength, and *N* represents the noise. For a given MEG dataset, *B* is known and *L* can be computed for each node with a forward solution. The forward solution in this study was computed according to Sarvas' formula for outside hemispherical conductors in Cartesian coordinates (Sarvas, 1987).

The determination of source strength and orientation of *Q* has been a challenge as discussed in many previous reports (Mosher et al., 1999; Huang et al., 2004; Robinson, 2004; De Munck and Bijma, 2009; Ou et al., 2009). According to our tests, the determination of MEG data in both low- and high-frequency ranges with conventional beamforming required considerable time and computing power to decompose MEG sensor data to subspaces because the data in both low- and high-frequency ranges had more data points as compared with the previous reports typically focusing on a single frequency range. However, for a given MEG data set in multiple frequency ranges in a limited time window (2 min in this study), the positions of sensor array and the 3D source grid were fixed; consequently, lead fields could be computed once and then used for both low and high-frequency ranges. Under these assumptions, we propose using SVD to decompose the lead field as following:

(6) $$L=USV^{T}$$

Where *U* ∈ *R*^*mxm*^ is an orthogonal (unitary in the complex case) matrix. The columns of U are the left singular vectors of L. *V* ∈ *R*^*mxm*^ is an orthogonal (unitary in the complex case) matrix. The columns of *V* are right singular vectors of *L*. *S* = *diag*(σ_1_, σ_2_,…σ_*p*_) is an *M* × *N* diagonal matrix with *p* = (*m*, *n*) and σ_1_, σ_2_,…σ_*p*_ are the singular values of *L*. *M* indicates the number of sensors and *N* indicates the number of source orientations. For a single source, *p* = 3. The Moore-Penrose pseudo inverse of *L* is given by:

(7) $$L^{+}=VS^{+}U^{T}$$

Where *S*^+^ is a diagonal formed with the multiplicative inverses of the nonzero singular values of *L* placed on the diagonal. Assuming there was no noise (*N* = 0), the measured MEG data, *B*, can be described by the following equations:

(8) $$B=LQ=USV^{T}Q$$

(9) $$Q=BL^{-1}$$

By replacing *L*^−1^ in Equation (9) with *L* in Equation (8), the estimated moment, $\vec{Q}$, can be computed with a SVD back substitution as described in the following equation:

(10) $$\vec{Q}=BVS^{+}U^{T}$$

Of note, *L*^+^, pseudo inverse of *L*, could be computed once and used for the analysis of data in all frequency ranges, which makes the computation of source strength and probability more efficient. In addition, once the $\vec{Q}$ is determined, virtual sensor spectrograms can be also computed with $\vec{Q}$ for each frequency range and time window.

(11) $$V(t,\;f)=\sum_{t=1}^{T}\sum_{f=1}^{F}||\vec{Q}|{|}_{2}{\left(TF\right)}^{-1}$$

In Equation (11), *V* represents the computed virtual sensor spectral data. The *t* and *T* indicate time slice and total number of time windows, respectively. The *f* and *F* indicate frequency band and total number of frequency bands, respectively. Magnetic signals generated by $\vec{Q}$ can be computed with the follow equation:

(12) $$X_{cmp}=L\vec{Q}$$

where *Xcmp* represents computed magnetic signals at individual sensors from source $\vec{Q}$. We used *Xmea* to represent the measured magnetic signals at individual sensors, which were different from *B* in Equation (3), which represents MEG data in general.

### Reliability assessment of source activity at low- and high-frequency ranges

To minimize the “ill-posed” inverse problem in MEG, the theory that a given MEG sensor data pattern may have an infinite number of possible “correct” answers (Hamalainen and Sarvas, 1987; Sarvas, 1987), we developed a channel-cross-channel (CxC) function to analyze the spatial pattern of MEG signals. Building on the use of covariance matrix for MEG beamforming in our previous studies (Kotecha et al., 2009; Gummadavelli et al., 2013), we applied a subtraction operation to all possible channel-pairs to generate a matrix which described the spatial gradient of magnetic signals among the sensors. Mathematically, each entry outside of the main diagonal in a CxC matrix represents the difference of a channel-pair. The diagonal entries represent the values of the corresponding sensors. To assess the reliability of source activity, the similarity of the measured MEG signal (*Xmea*) and the computed MEG signals (*Xcmp*) were statistically analyzed with the CxC matrix by computing the covariance and correlation factors with the following formulas:

(13) $$C\text{​}\left(x_{mea},\;x_{cmp}\right)=\frac{\sum_{i=1}^{K}\left(x_{mea}i-\bar{x_{mea}})(x_{cmp}i-\bar{x_{cmp}}\right)}{N-1}$$

(14) $$R\text{​}\left(x_{mea},\;x_{cmp}\right)=\frac{C(x_{mea},\;x_{cmp})}{Sx_{mea}Sx_{cmp}}$$

Where *C* (*x*_*mea*_, *x*_*cmp*_) indicates the covariance and *R* (*x*_*mea*_, *x*_*cmp*_) indicates the correlation in the CxC matrices. The *x*_*mea*_ and *x*_*cmp*_ indicate signals in two channels which were paired for computing CxC. *x*_*mea*_ and *x*_*cmp*_ represent the mean of the signals in the measured and computed datasets, respectively. Sx_*mea*_ and Sx_*cmp*_ indicate the standard deviation of the signals in the two datasets, respectively. *K* indicates the number of sensors used for source estimation, which was smaller or equal to the total number of measuring sensors. To statistically determine the spatial correlations for each node in the 3D grid, *t*-values were computed for all sources.

(15) $$Tp=R\sqrt{\frac{K-2}{1-R^{2}}}$$

In Equation (15), *Tp* is the *t*-value of a source; *R* indicates the correlation of the measured and computed MEG signals for the source; *K* indicates the number of sensors related to the source.

A careful observation of Equation (13) could find that *x*_*cmp*_ is similar to the weights of the conventional beamforming because *x*_*cmp*_ represents signals from a predefined location and estimated source orientation. Similar to the conventional beamforming, the use of *x*_*cmp*_ could maximize signals from the source and minimize environmental noise and signals from other locations. For the analyses of multi-frequency signals, the location-orientation weights were computed from the optimal CxC matrix for each frequency. Thus, the source orientation was independent of frequency and only dependent on the orientation of the cortical normal vector. In other words, the solutions are approximations; the orientation portion was frequency independent.

Building on previous reports that the spectral signatures of low- and high-frequency signals at source levels can be measured with the combination of accumulated spectrogram and virtual sensors (Xiang et al., 2004, 2009a,b; Xiang and Xiao, 2009), the present study developed accumulated source imaging (Figure 2). With this technique, an accumulated source image was generated by accumulating all the source data computed for each location and each frequency band from the entire epoch of the MEG data. Of note, the computing of accumulated source images maintained spatial- and frequency-locked signals and minimized signals in random-space and frequency.

### Magnetic source imaging with multi-parameters per location (MPPL)

This study moved one step further by developing magnetic source images with multi-values per location or MPPL. Specifically, each location has multi-parameters: (1) the first parameter describes the frequency range, which is represented with a frequency index for minimizing the use of computer memory and storage spaces; (2) the second parameter describes the strength of source activity; (3) the third parameter describes the reliability of the source; (4) the fourth parameter describes the Kurtosis or “peakedness” of source activity. The frequency index was directly obtained from the processed MEG data (the values of high-pass and low-pass filters or the frequency index in time-frequency representation). The strength of source activity was the source moment computed with Equation (10). The reliability could be computed with Equations (14) or (15). Building on previous report (Robinson et al., 2004), the Kurtosis was computed with following equation.

(16) $$K=\frac{\sum_{t=1}^{T}{\left(q(t)-u\right)}^{4}}{T\sigma_{t}^{4}}-3$$

Where *T* is the length of source data *t* in a time window, which has a mean of *u* and a standard deviation of σ. *K* represents the kurtosis values and is stored in parameter 4 in accumulated source imaging.

As shown in Figure 2, the analyses of MEG signals at both low- and high-frequency ranges generated more than one value for each location or each node of the 3D grid (e.g., strength, reliability and frequency of source activity). Notably, conventional magnetic source imaging, which encodes one value for one location or voxel, cannot represent the source data computed with the developed methods. The main differences between the new methods and existing methods are summarized in Table 1.

**Table 1:** **Differences between accumulated source imaging (ASI) and similar methods**.

	**ASI**	**DM**	**SAM**	**SAM(g2)**	**BF**	**MN**	**MUSIC**
Optimized for localizing HFOs	Yes	No	No	No	No	No	No
Handle large dataset	Yes	No	No	No	No	No	No
Handle multi-frequency signals	Yes	No	No	No	No	No	No
Multi-parameter per location	Yes	No	No	No	No	No	No
Volumetric source scan	Yes	No	Yes	Yes	Maybe	Yes	Yes
Detect dynamic sources	Yes	Yes	No	No	No	Yes	Yes
Detect stationary sources	Yes	No	Yes	Yes	Yes	No	No
Detect correlated sources	Yes	Yes	No	No	No	Yes	Yes
Noise suppression	Yes	No	Yes	Yes	Yes	No	Yes

*ASI, accumulated source imaging; DM, dipole modeling (dipole fitting); SAM, synthetic aperture magnetometry; SAM (g2), SAM excess kurtosis (g2); BM, conventional beamforming; MN, minimum-norm; MUSIC, multiple signal classification*.

### Source localization with accumulating

Accumulated source imaging was defined as the volumetric summation of source activity over a period of time which was at least two times longer than that of the time window of the source image. Of note, accumulated source imaging could have more than 1 time slices to reveal the fluctuation of source activity in space and time. Accumulated source imaging can be described as the following equation:

(17) $$Asi(r,\;s)=\sum_{t=1}^{t=n}Q(r,\;t)$$

In Equation (17), *Asi* represents accumulated source strength at location *r*; *s* indicates the time slice; *t* indicates time point of MEG data; n indicates total time points of MEG data and *Q* indicate the source activity at source *r* and at time point *t*. We defined that *s* ≥ 1 and *s* ≤ *n*/2. From a computer program point of view, the use of computer memory and storage space by Equation (12) is dependent on the *s* for a fixed source imaging configuration (e.g., spatial resolution and dimension). Even though *n* could be infinitely increasing, the requirements for computer memory and storage remain the same. Consequently, the approach automatically avoided possible “overflow” or “out of space” problems in a long-time or continuous recording for capturing epileptic activity such as spikes. Since accumulated source imaging accumulates the results of source data, it is different from previous reports which compute a covariance matrix or kurtosis of sensor data for a long-time recording. Specifically, using a covariance matrix or kurtosis computed with sensor data for a long-time recording for source localized is based on the assumption that the source was stationary during the long-time recording. Our approach, on the other hand, did not make this assumption. Therefore, our approach has the capability to detect both stationary and nonstationary source activity.

### MEG experiments, MRI scan and intracranial recordings

#### Participants

Ten healthy children (5 girls; 5 boys; age: 6–18 years; mean age: 12.8 years) were recruited for this study. Inclusion criteria were: (1) healthy without a history of neurological disorders or brain injuries; (2) age-appropriate functions including hearing, vision, and hand movement; (3) head movement during MEG recording was less than 5 mm. Ten pediatric patients (5 girls; 5 boys; age: 6–18 years; mean age: 12.7 years) with clinically diagnosed epilepsy were retrospectively studied. Patient inclusion criteria were: (1) clinically diagnosed epilepsy; (2) head movement during MEG recording was less than 5 mm; and (3) epileptic foci were confirmed with electrocorticography (ECoG) and/or neuroimaging data. Exclusion criteria were: (1) inability to remain still; and (2) presence of an implant such as a cochlear implant device, a pacemaker, or a neuro-stimulator containing electrical circuitry, generating magnetic signals, or having other metal that could produce visible magnetic noise (>6 pT) in the MEG data. Written consent, formally approved by the Institutional Review Board (IRB) at Cincinnati Children's Hospital Medical Center (CCHMC) and Nanjing Brain Hospital, was obtained from each healthy participant prior to testing. This study was approved by IRB at CCHMC.

#### MEG recordings

MEG signals were recorded in a magnetically shielded room (MSR) using a whole head CTF 275-Channel MEG system (VSM MedTech Systems Inc., Coquitlam, BC, Canada) in the MEG Center at CCHMC. Before data acquisition commenced, three electromagnetic coils were attached to the nasion, left and right pre-auricular points of each subject. These three coils were subsequently activated at different frequencies for measuring each subject's head position relative to the MEG sensors. Each subject lay comfortably in the supine position, his or her arms resting on either side, during the entire procedure. MEG data were recorded at a sampling rate of 4000 Hz. Continuous MEG recordings were completed for an epoch of 2 min. To ensure the reproducibility, at least two epochs were recorded for each subject. All MEG data were recorded with a noise cancellation of third order gradients and without on-line filtering. To identify system and environmental noise, we routinely recorded one MEG dataset without a subject immediately prior to the experiment.

#### MRI scan

Three-dimensional magnetic resonance imaging (MRI) was obtained using a 3-T Philips Achieva scanner (Philips Healthcare, Andover, MA). Three fiduciary marks were placed in identical locations to the positions of the 3 coils used in the MEG recordings with the aid of digital photographs to allow for an accurate co-registration of the 2 data sets. Subsequently, all anatomic landmarks were made identifiable in the MRIs.

Similar to previous reports (Xiang et al., 2009a), clinical intracranial electrocorticography (ECoG) data were retrospectively analyzed with the MEG results. Of the 10 patients, the 8 patients reported here had implantation of subdural electrodes and CCTV/EEG (VEEG) monitoring according to standard protocol at our hospital. Digital photos were taken before and during the operation to record the placements of the electrodes.

### Implementation of the algorithms

The aforementioned method for reconstruction of brain activity was implemented in MEG Processor with C/C++ on Windows platform (Xiang et al., 2010; Gummadavelli et al., 2013). MEG Processor was driven by its Windows interface. From the user perspective, its organization is contextual rather than linear: the multiple features from the software were not listed in long menus, they were accessible only when needed and were typically suggested within contextual popup menus or specific interface windows. This structure provided faster and easier access to requested functions.

### Data analyses

MEG data were visually inspected for artifacts. MEG waveforms with identifiable artifacts (amplitude >6 pT) were excluded from data analyses. Similar to previous reports (Xiang et al., 2009a), accumulated spectrograms, global spectrograms and spectral contour maps for all subjects were computed and analyzed. Before reconstructing brain activity for human MEG data, the head was modeled as a homogenous conducting sphere in order to account for volume-conducted return currents. The sphere model used in this study was a multiple local-sphere model, where each sphere (one per MEG sensor) was fit to a small patch of the head model (directly under the sensor) in order to better model the local return currents (Huang et al., 1999). The conducting boundary was defined with individual MRI, which was the inner skull. In other words, the best-fit sphere was fit to the scalp. From this head model, a whole-brain, subject-specific lead field was computed and used for magnetic source reconstruction. Accumulated source imaging and conventional beamforming (Vrba and Robinson, 2001) were implemented in MEG Processor for source estimation (Kotecha et al., 2009; Chen et al., 2010; Gummadavelli et al., 2013). CTF software package (VSM MedTech Systems Inc., Coquitlam, BC, Canada) was used to perform dipole fit analyses (Robinson et al., 2004; Kirsch et al., 2006). We used MNE (Gramfort et al., 2014) and Brainstorm (Tadel et al., 2011) to perform source estimation with Minimum-norm and multiple signal classification (MUSIC) algorithms, respectively.

To quantify the results, electrocorticography (ECoG) was used as the “gold standard” for defining epileptic zones. MEG sources were overlapped onto individual MRI data. Cerebral landmarks including the central sulcus, Sylvian fissure and the somatosensory cortex were used to define specific anatomical cortical brain regions (Agirre-Arrizubieta et al., 2009). The brain regions were the central, parietal, and occipital lobes. The frontal lobe was divided in the frontal superior, medial, inferior, and fronto-orbital regions; the temporal lobe into the lateral and mesial regions, the latter comprising the amygdala, the hippocampus, the parahippocampal gyrus, and the temporal-basal area. The inter-hemispheric region consisted of the mesial surface of the frontal, parietal, and occipital lobes (De Gooijer-Van De Groep, 2013). Similar to previous reports (Agirre-Arrizubieta et al., 2009; De Gooijer-Van De Groep, 2013), the concordance between MEG sources and ECoG was measured by determining if the interictal ECoG and MEG source locations were anatomically matched in the brain regions. We defined the sensitivity and specificity of the methods as followings.

(18) $$Sensitivity=\frac{TP}{TP+FN}$$

(19) $$Specificity=\frac{TN}{TN+FP}$$

Where TP represents the number of true positive (both MEG and ECoG showed epileptic foci); FN indicates the number of false negative (ECoG showed epileptic foci while MEG showed no epileptic focus); TN represents the number of true negative (both MEG method and ECoG showed no epileptic foci); FP represents number of false positive (MEG showed epileptic foci, but ECoG showed no epileptic focus).

### Statistical analysis

The comparisons of spectral and source data for epilepsy subjects and controls were performed with paired Student *T*-tests. The odds ratios of activity in brain areas in epilepsy subjects other than the areas identified in control groups for each frequency band were analyzed with Fisher's exact tests. Significance was accepted at the level of *p* < 0.05 for one comparison. Since multiple frequency bands and more than one source were analyzed, Bonferroni multiple comparison corrections were applied. Specifically, if multiple comparisons were to be taken into account then the significance level for any one of these comparisons was reduced from 0.05 to 0.05/parameter (e.g., for 9 frequency bands, *p* < 0.005).

## Results

**Figure 3:**
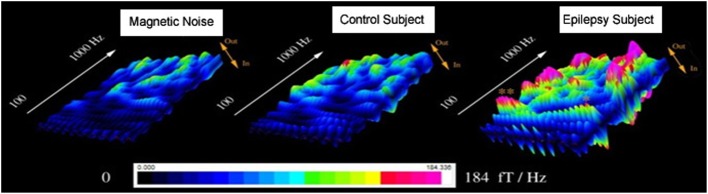
**Accumulated global spectrograms in three conditions**. “Magnetic Noise” was computed with MEG data recorded without subjects. “Control Subject” was computed with MEG data recorded from a healthy child. “Epilepsy Subject” was computed with MEG data recorded from a child with epilepsy between seizures (interictal). The sampling rate of all MEG recordings was 6000 Hz. An accumulated global spectrogram represents the “spatial summation” of the entire MEG sensor array accumulated spectrograms. The three spectrograms show that the epilepsy subject has elevated spectral power as compared to the control subject.

**Figure 4:**
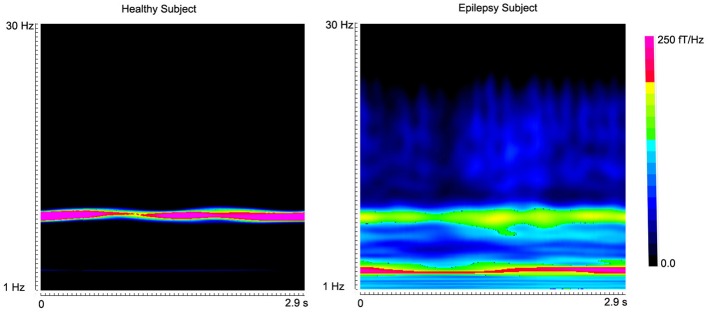
**Accumulated spectrograms show the well-known alpha activity in a healthy subject and an epilepsy subject**. Noticeably, healthy subject (“Healthy Subject”) has a clear activity around 8–12 Hz (alpha activity). However, the epilepsy subject (“Epilepsy Subject”) has incrased activity in 2–4 Hz (low-frequency activity). The color bar shows the color coding of spectral power.

**Figure 5:**
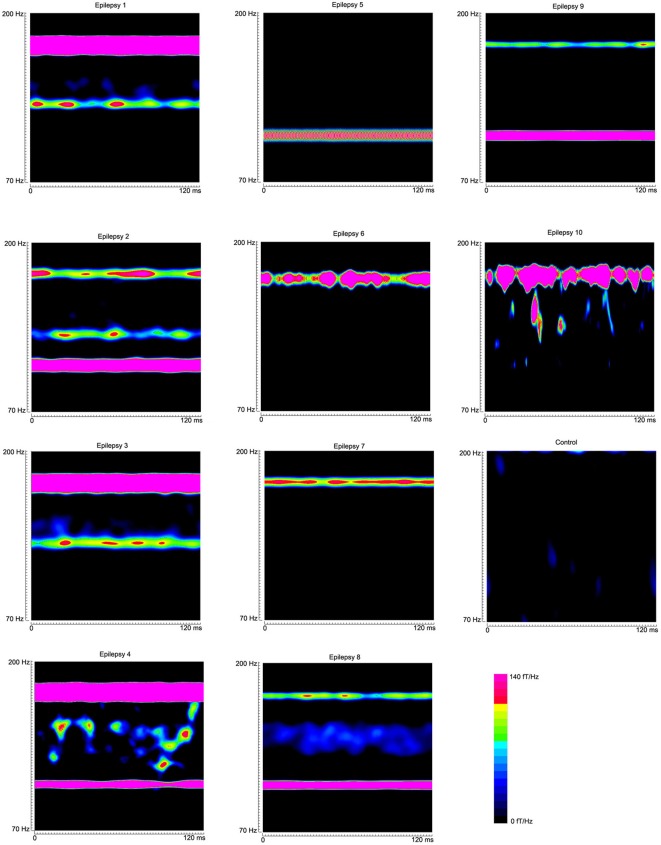
**Global accumulated spectrograms from 10 epilepsy subjects and 1 healthy subject show the main frequency components of neuromagnetic signals in 70–200 Hz in epilepsy patients**. Noticeably, the activity patterns vary across patients. The color bar shows the color coding of spectral power for all the global accumulated spectrograms.

**Figure 6:**
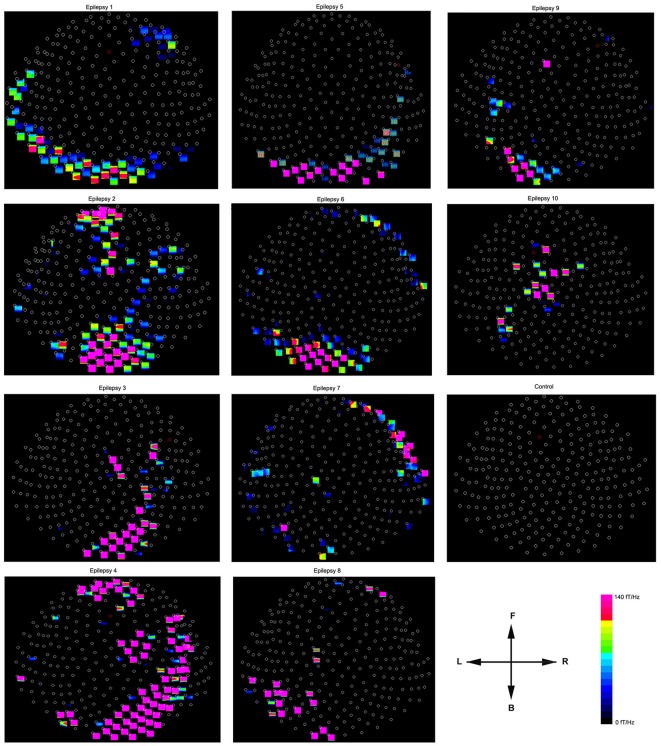
**Accumulated spectral contour maps from 10 epilepsy subjects and 1 healthy subject show the spatial distributions**. Noticeably, the spectral distribution varies across patients. All the contour maps have the same orientation defined by the arrows: the “L” indicates the left side of the head and the “R” indicates the right side of the head. The “F” indicates that the upper part of the contour map represents the frontal region of the head; the “B” indicates that the lower part of the contour map represents the posterior region of the head. Each small circle represents one MEG sensor. The color bar shows the color coding of spectral power for all the contour maps.

The size of 2 min MEG data digitized at a sampling rate of 4000 Hz (CTF MEG system, 275 sensors) was 0.597 GB. For time-frequency analyses, if the frequency bin of time-frequency transform was 600, the size of the time-frequency representation of 0.597 GB waveform data were 358 GB (600 × 0.597). When we computed the CxC data with time frequency data, the size of time-frequency based covariance matrices were 128164 GB (358 × 358 GB), which was approximately 125 TB. Of note, the source data computed from the time-frequency data would also be larger (>125 TB). Since the physical memory limit for windows 7 (64 bits, professional version) was 192 GB, the spectral data computed with the conventional time-frequency analysis method could not be stored in our Windows workstations because as it clearly exceeded the upper limits of the operating system. Alternatively, with accumulated spectrogram, we were able to limit the size of the spectrogram to 3 GB. Noticeably, the size and time required for computing an accumulated spectrogram mainly depended on the dimension of the accumulated spectrogram (number of frequency bins and time slices) and frequency ranges which could be adjusted by users. For an accumulated spectrogram with a dimension of 600 × 600 (600 frequency bins, 600 time slices) for a 2 min recording (sampling rate 4000 Hz), it took approximately 8.1 ± 0.03 h for data in 70–200 Hz, 1.3 ± 0.002 h for data in 1–70 Hz. Of note, the processing time would also depend on the speed of CPU and GPU, the number of programs running, the optimization of software compiling. In this study, we used two CPU (Intel Xeon, E4506, 2.13 Hz, each CPU has four cores). If GPU was used, the times were shortened to 42.5 ± 0.31 min for data in 70–200 Hz and 12.2 ± 0.009 min for 1–70 Hz, respectively. GPU could significantly shorten the computing time (*p* < 0.0001). Examples of accumulated spectrograms are shown in Figures 3–6. To identify high-frequency signals in multiple frequency bands with visual inspection, it took 2–3 days for a neurologist with 8 years of EEG/MEG experience.

The processing time for source scan with the conventional dynamic multi-dipole modeling (finding the 13 dipole for each time-slice) in multi-frequency ranges for recording at a sampling rate of 4000 Hz took 92.3 ± 0.4 h. However, our accumulated source imaging, which automatically scanned the entire brain for the same dataset took 12.7 ± 0.4 h. Of note, the approach was approximately 7.6 times faster than the conventional approach (*p* < 0.0001). If GPU was used, the time could be significantly shortened to approximately 6.3 ± 0.1 h. However, the use of GPU slowed down the user responses in our tests.

The global spectrograms of MEG datasets recorded from three conditions (no subject, healthy subjects and epilepsy subjects) showed that the epilepsy subjects had significantly increased spectral power. Figure 3 shows an example of global spectrograms in the three conditions. We noted that accumulated spectrograms revealed a clear alpha activity (approximately 8–12 Hz) in all healthy subjects (10/10, 100%) (Figure 4). Out of the 10 epilepsy patients, 9 patients showed increased spectral power in 70–200 Hz (9/10, 90%). Further analyses revealed that increased spectral power were around 106, 140, and 168 Hz in epilepsy patients (Figure 5). Figure 6 shows the spatial distributions of accumulated spectrograms in spectral contour maps.

**Figure 7:**
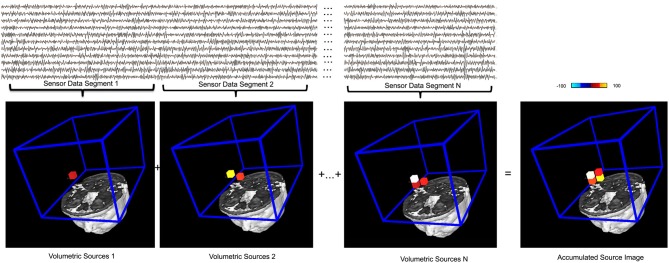
**An illustration of the basic principle of accumulated source imaging**. The top waveforms show MEG data at sensor levels. The bottom images show individual structural magnetic resonance image and the region of interest (ROI, blue lines) for source scanning. MEG sensor data are firstly divided into small segments (e.g., “Sensor Data Segment 1,” “Sensor Data Segment 2,” “Sensor Data Segment N”). Volumetric sources are then produced by scanning the entire ROI with each segment of sensor data. The red, yellow and white small cubes indicate the sources (or voxels) identified. For illustration purposes, a very low resolution (12 millimeter) spatial resolution was used. An accumulated source image is generated by spatially adding all volumetric sources together. Of note, only sources reach certain thresholds (in this case, 75%) are added to accumulated source images, which differentiate this accumulating process from averaging. The color bar indicates the color coding of the source strength.

**Figure 8:**
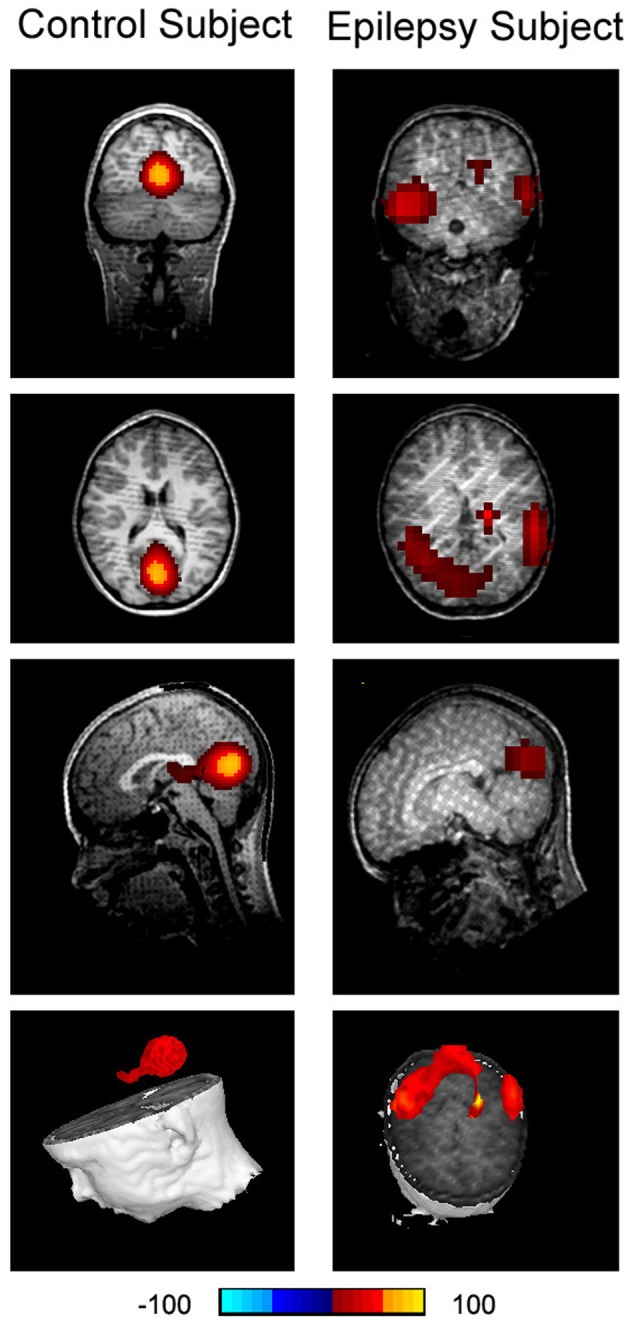
**Accumulated source imaging shows low frequency brain activity in 8–12 Hz (alpha) in an epilepsy subject (“Epilepsy Subject”) and a healthy subject (“Control Subject”)**. Alpha activity is localized to the occipital cortex in the healthy subject. However, alpha activity is overshadowed by epileptic activity in the epilepsy subject. The epileptic activity is localized to the left and right parietal cortices in the epilepsy subject, which is concordant with clinical findings.

Accumulated source imaging revealed focal increase of spectral power (Figure 7). Accumulated source imaging in low frequency ranges revealed that brain activities in 8–12 Hz (alpha) were localized to the occipital cortex in all the healthy subjects (10/10, 100%). However, alpha activity were localized to the occipital cortex in five epilepsy patients (5/10, 50%) and in nonoccipital cortices in other five patients (see Figure 8 for example). The five patients all had strong epileptic activity, which overshadowed and/or interrupted alpha activity. We noted that the increased spectral power at source levels varied among epilepsy patients.

**Figure 9:**
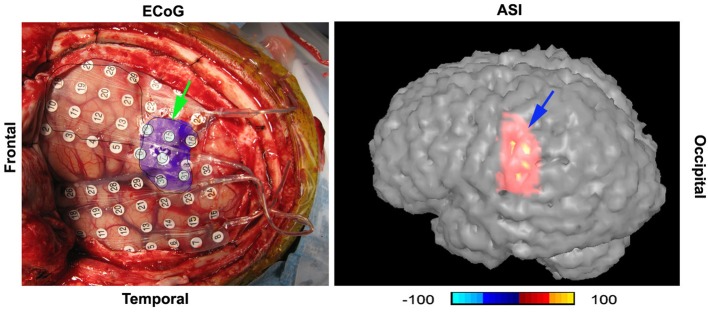
**A digital photo of intracranial recording (“ECoG”) and accumulated source imaging (“ASI”) show the concordance of the two methods**. The two images are placed in the similar orientation. “Frontal” indicate the frontal cortex; “Temporal” indicates temporal lobe. The left green arrow points to the epileptic area invasively defined by intracranial recording; the right blue arrow points to the epileptic area noninvasively localized with high-frequency neuromagnetic signals. Noticeably, the areas are matched in gyrus level. The color bar shows the color coding of accumulated source imaging. The value of the source voxel is normalized T value (no unit).

Accumulated source imaging showed that 9 out of the 10 epilepsy patients (9/10, 90%) had increased focal spectral power in high-frequency ranges at source levels. The epileptic areas localized by accumulated source imaging were concordant with clinical data. Figure 9 shows an example of epileptic foci volumetrically localized with high-frequency accumulated source imaging (70–200 Hz). The sensitivity and specificity of all subjects are shown in Table 2.

**Table 2:** **The sensitivity and specificity of five MEG source localization methods**.

		**ASI**	**DM**	**BF**	**MN**	**MUSIC**
1–70 Hz	Sensitivity	72	50	60	70	70
	Specificity	64	38	59	60	54
70–200 Hz	Sensitivity	90	30	40	50	40
	Specificity	76	42	53	62	68

## Discussion

The present study demonstrated an approach for detecting both low- and high-frequency neuromagnetic signals by integrating time-frequency transform, source localization, accumulation and MPPL algorithms into a comprehensive and systematic processing package. The strengths of our methodologies are reflected by the major features of our signal processing algorithms as well as their abilities to resolve the difficulties associated with the large data volume, multi-modality data and its clinical applicability.

### Features of our signal processing algorithms

One of the unique features in our wavelet transform algorithm was that the sigma value (number of waves) could be dynamically changed so as to match the neurophysiological patterns. For example, neuromagnetic signals from a brain area may appear in multiple frequency ranges but in a similar time window. The conventional wavelet algorithm typically gives a wide time-window for low signals and a narrow time-window for high-frequency signals, which is not well-suited for analysis of brain activity. The improved wavelet transform algorithm in the present study could solve this problem by dynamically changing the sigma values so as to adjust the time-window for a better analysis of brain activity.

Accumulating algorithms in the computing of accumulated spectrograms provides a novel method for handling the large datasets obtained when analyzing both low and high-frequency MEG signals. Integration of time-frequency analysis and accumulation into a workflow system is a novel neuroimaging data processing algorithm technique that can summarize and visualize high-frequency signals with a few images.

CxC matrices and functions are critical to the study of neural HFOs. Since high-frequency signals are typically obscured by low-frequency signals (Xiang et al., 2009a), time-frequency representations were normalized according to the magnitude of each frequency bin across all MEG sensors to ensure that all frequency bins contributed equally to the source reconstruction. The time-frequency matrix allows for matrix operations such as subtraction of control state MEG signals from the activation state MEG signals, whose purpose is to increase signal-to-noise ratio (SNR) or to maximize the signal power at a peak frequency (or a frequency of interest) while simultaneously minimizing it at the neighboring surrounding frequency bins. CxC matrices based on the time-frequency data provide unique spatial patterns and gradients of magnetic fields for determining high-frequency sources.

The major differences between our technique and existing methods of volumetric imaging such as beamforming, minimum-norm are the features of accumulation and MPPL, which are more than a source localization algorithm. To our knowledge, none of the existing methods have the features of accumulation and MPPL. It is necessary to point it out that, some existing methods have internally fixed frequency ranges (e.g., 20–70 Hz) (Robinson et al., 2004), which could not be directly compared in our tests because our method was designed to analyze both low- and high-frequency signals (multi-frequencies). Of note, each method has its strengths and weaknesses (De Gooijer-Van De Groep, 2013). According to on our clinical experience, the unique features of the method are clinically important and necessary. For example, the conventional beamforming could also been used to detect multi-frequency signals (Vrba and Robinson, 2001). However, the conventional beamforming is based on covariance matrices, which are computed from data in long time-windows (if the time-windows are short, the sizes of the source data would be a problem). The process assumes that the brain activity is stationary in the time-window (Vrba and Robinson, 2001), which may be not true for real epileptic activity (Zijlmans et al., 2012b). By using accumulation algorithms, our approach does not making any assumption about the stationarity of the sources. Another example is SAM (g2). SAM (g2) is an outstanding method for detecting excess kurtosis (Kirsch et al., 2006). SAM (g2) is designed for detecting rare events (spikiness activity). It has been shown that combining SAM(g2) and other methods such as MUSIC gives the best clinical results (De Gooijer-Van De Groep, 2013). The development of MPPL in our method enables us to implement both kurtosis and other algorithms by using multiple parameters during source analyses. Consequently, both rare events (kurtosis) and common events (frequent spikes) could be detected by our methods.

### Several major challenges resolved with the current methodologies

#### Data volume challenge

Our results are consistent with previous reports (Blanco et al., 2011), the size of high sampling rate MEG/EEG data could be in the magnitude of TB (>12 TB). This was particularly true for multi-frequency spectral data (>125 TB). However, by using accumulating techniques, we were able to minimize the size of MEG data to less than 10 GB without losing the high-frequency information. Although accumulated spectrogram was utilized with MEG in the present study, the same technology can also be used in the analysis of EEG and intracranial EEG. In current clinical research, the detection and labeling of interictal and ictal epileptiform activity in intracranial EEG recordings is performed by expert review. This manual method has been known to be associated with a poor inter-reviewer reliability (Benbadis et al., 2009). In addition, manual review is not feasible for large data sets because it is very time consuming and labor-intensive (Restuccia et al., 2011; Andrade-Valenca et al., 2012; Dumpelmann et al., 2012; Jacobs et al., 2012; Zijlmans et al., 2012a; Haegelen et al., 2013; Stacey et al., 2013). Alternatively, accumulated source imaging can automatically analyze large data sets and provide images for experts to review. This new method can be used in combination with experts' review to verify its sensitivity and specificity and to advance our understanding of the relationship between HFO and epilepsy. According to our data, the new method can be further developed as a fully automatic detector with high specificity and sensitivity.

The development of methods for analysis of a substantial amount of MEG/EEG data has become an important research area. For example, data mining has been developed for the analysis of HFOs in epilepsy patients (Blanco et al., 2011; Worrell et al., 2012). Blanco et al. (2011) reported a quantitative analysis of HFOs and their rates of occurrence in 9 patients with neocortical epilepsy and two control patients with no history of seizures (sampling rate: 32,556 Hz). Using the data mining approach, they found that a cluster of ripple frequency oscillations with a median spectral centroid of 137 Hz is increased in the seizure-onset zone more frequently than a cluster of fast ripple frequency oscillations (median spectral centroid = 305 Hz). Our results are consistent with their findings. The relative rate of ripple frequency oscillations is an interesting potential biomarker for the epileptic neocortex, but larger prospective studies correlating HFOs rates with seizure-onset zones, resected tissue and surgical outcomes are required to determine the true predictive value of this line of research (Montazeri et al., 2009; Blanco et al., 2011; Worrell et al., 2012). However, to our understanding, algorithmic requirements differ substantially for data mining and for topological (feature) data analysis. In particular, little is known about the locations of high frequency brain signals and their relationship to neurological disorders. In this regard, one of the unique features of accumulated source imaging is its ability to localize and visualize epileptic activity in both low- and high-frequency ranges for correlating locations of brain signals to neurological disorders.

#### Multi-modality imaging data challenge

Our data have also shown that functional MEG data can be seamlessly integrated into structural MRI data. In comparison to conventional source imaging, one important feature of accumulated source imaging is MPPL. MPPL analysis results in multi-values per voxel in 3D images. One parameter is dedicated to the frequency signature, which is important for visualizing HFOs. For example, HFOs may be a band-limited event (Crepon et al., 2010) or they can be a broadband event (Staba and Bragin, 2011; Worrell et al., 2012; Zijlmans et al., 2012b). By visualizing the frequency in the imaging data, we can better address many current questions in the study of HFOs (Engel et al., 2009; Staba and Bragin, 2011; Worrell et al., 2012; Zijlmans et al., 2012b). For example, if HFOs are band-limited, should there be specific spectral boundaries? In other words, should a HFO be defined as an isolated event in the time–frequency map, or could it contain a variety of frequencies within a range? Since spontaneous activity can occur in multi-frequency ranges, we consider the development of accumulated source imaging with MPPL to be important for multi-modality integration because the unique parameters from MEG is encoded in each location (voxel) and can be easily integrated into other modalities without losing any information.

Integration of accumulating and source localization into a systematic approach is a powerful neuroimaging data processing technique that could simplify multi-modality analyses. For example, epileptic foci defined by HFOs are not time-locked and can spontaneously occur at any time point or time window. Without accumulation, thousands of MEG source images may need to be integrated into structural MRIs, which would be time-consuming. With accumulated source imaging, epileptic foci can be captured and summarized as a few images, which could be easily integrated into structural MRIs. As demonstrated in the Results section, accumulated source imaging is a potential powerful technique for multi-modality analysis of epileptic foci. Importantly, our software packages and libraries are based on C/C++. This methodology can be similarly implemented in more advanced computer systems such as cluster/GPU/FPGA or cloud/HPC. By using those advanced computer technologies, accumulated source imaging can be computed in a timely manner and routinely used in clinical practice in the future.

#### Clinical applicability challenge

Building on previous reports (Robinson et al., 2004; Kirsch et al., 2006) and our clinical observation, we developed the aforementioned method for detecting both low- and high-frequency brain signals. The proposed framework and architecture may also solve a few problems occurring in our clinical practice. First, this method provides an objective means of data analysis. The existing and currently practiced method for identifying epileptic spikes relies on visual inspection, which is subjective. Second, the proposed method provides meaningful quantitative source data, which are not available in conventional visual identification of epileptic spikes. Third, the new method can provide novel frequency descriptions about aberrant brain activity. In addition, the newly developed method semi-automatically quantifies MEG spectral power and source activity. Moreover, the new method has the capability of detecting and localizing high-frequency epileptic signals, a feat impossible to achieve with the conventional visual inspection of waveforms.

The results of spectral data showed that accumulated source imaging may play a key role in the differentiation of true HFOs from environmental noise in pre-operative workup for epilepsy surgery. It is well known that low-frequency signals may generate high-frequency harmonics. Since any harmonic of a high-frequency signal will localize to the corresponding low-frequency component, accumulated source imaging (which encodes both frequency and spatial information) can automatically reveal the main frequency by comparing the spectral power in the location of question. If HFOs were localized to a brain area which did not have low-frequency signals, the location would be an index for true HFOs. Since digital filtering may be used in the analysis of HFOs, the filter characteristics must be taken into account to avoid the detection of false oscillations (Benar et al., 2010). It has been noted that sharp transients with spectral content in HFO bands but without actual HFO in the raw data may be generated by filtering. According to our observation, such false oscillations are typically the result of the additive superposition of harmonics. They do not have a consistent spatial pattern in CxC and cannot be consistently localized to a location in the brain.

Accumulated source imaging may also play a key role in the differentiation of brain HFOs from artifacts in clinical practice. Raw MEG data contain a mixture of high-frequency brain signals and a variety of artifacts and noise. A major obstacle to HFO research is the unfortunate fact that various muscle activities typically result in prominent increases in gamma power (>25 Hz), and contaminate the recorded signal in the HFO spectrum. Myogenic activity interferes with the detection of HFO and represents a significant and often under-estimated challenge in clinical and basic research. For many years, intracranial EEG recordings were assumed to be largely, if not completely, immune to eye movements and muscle artifacts. This assumption has recently been proven to be erroneous (Ball et al., 2009; Jerbi et al., 2009; Kovach et al., 2011). To solve these problems, we tried a different approach. Since high-frequency signals are typically obscured by low-frequency signals, time-frequency representations could be normalized according to the magnitude of each frequency bin across all MEG sensors to ensure that all frequency bins contributed equally to the source reconstruction. The time-frequency matrix allows for matrix operations to maximize the signal power at a peak frequency while simultaneously minimizing it at the neighboring surrounding frequency bins. CxC matrices based on the time-frequency data provide spatial patterns and gradients of magnetic fields for accurately determining epileptic foci for clinical purposes. In addition, the method was able to show multiple metrics of source analyses. It is important to be able to visualize different metrics of the source data because the frequency, strength, reliability/probability and kurtosis are important for us to correctly interpret the results. According to our pilot data, very strong high-frequency sources (>100 Hz) typically pinpointed to the epileptogenic zones and the removal of these zones would likely result in good surgical outcomes and ultimately seizure freedom (Xiang et al., 2009a). Thus, we postulate that these multiple metrics of source data will allow discrimination among pathological, benign or artifactural source signals in the future.

Although our newly developed method showed promising results for detecting both low- and high-frequency brain signals, several weaknesses and problems have been identified and need to be addressed in the future. Specifically, we used multiple local spheres in the computation of forward solution, which did not address the effect of the inferior conductive boundary of the skull that is not proximal to any MEG sensors. This leads to questions as to the accuracy of the forward model for “deep” sources, as may be encountered in temporal lobe epilepsy. A model based on the superior and lateral curvature of the head may mitigate this problem. The Sarvas forward solution, as applied to each sensor's sphere origin could only compute the field due to the tangential components of the dipole moment. Given that there were multiple sphere origins that might be in the vicinity of one another, the dipole orientation and therefore the weights might be “confused” by the rapid change (with location) of the tangential orientation. The number of sensors in node-beam lead field was experimentally determined by using from 3 to 275 sensors. Since three sensors have only two degrees of freedom left to attenuate unwanted interference or brain signal, at least five sensors are necessary to discriminate between brain source and interference (with 3rd gradient compensation). Of note, we continue to perform research into improving our methodology to overcome some of these limitations.

We also noted that source activities in a few subjects were close to the brain-stem. Those activities might be an artifact or localization problem or real sources. According to our data, the activity in the center of the brain is more than likely real for several reasons: (1) MEG data recorded without subjects did not show similar sources. Thus, it is unlikely that the sources are from system artifacts (e.g., hardware, software or localization algorithms); (2) the shape of the volumetric sources appears to mimic the structure of individual structural MRIs in subjects. If the deep sources are system artifacts or localization problems, they should not mimic the structure of individual MRI. (3) There are reports showing that MEG can detect and localize source in the deep brain areas. (4) We were very careful to exclude artifact by excluding subjects with magnetic artifacts, recording MEG data with third-gradient noise cancellation and by visually inspecting the MEG data. However, MEG was not sensitive to sources in the brain areas and the source images of the deep source are more diffuse as compared to the surface sources. Therefore, further investigation and verification are necessary. We consider that closely following the guidance recommended by Gross and colleagues may further improve the quality of the data (Gross et al., 2013). In particular, by using multiple metrics of source analysis, we can incorporate new algorithms into data analysis by adding one source parameter. For example, Fatima and colleagues have developed a novel method to significantly improve the detection and localization of MEG sources by using independent component analysis (ICA) (Fatima et al., 2013), which can be incorporated into our source analysis pipeline to correct artifact and improve source localization. For resampling, one could also reduce the number of samples by high pass filtering the raw data and heterodyning it down to baseband, followed by decimation. The software and supplementary materials, which implemented the aforementioned algorithms, are freely available from the following website (http://sdrv.ms/PHenGK) for other researchers to test, reproduce, and improve the methods.

## Summary

In summary, the present study has demonstrated that accumulated source imaging is a new powerful technique for quantitatively and objectively analyzing MEG signals at source levels. By volumetrically scanning sources and accumulating source information, accumulated source imaging could handle very large datasets and extract meaningful spatial information about brain activity. Accumulated source imaging based on HFO detection may play a key role in differentiating brain activity from environmental noise and muscle artifacts. Though further verification is necessary, we believe that the next study should focus on using more advanced computer systems such as cluster/GPU/FPGA/cloud/HPC to significantly improve the performance of the proposed methods for clinical applications in the future.

## Author contributions

Jing Xiang: study design and methodological development. Hisako Fujiwara, Nat Hemasilpin, Douglas F. Rose: data acquisition. Abraham Korman, Fawen Zhang, Jing Xiang: data analysis and manuscript preparation. Qian Luo, Rupesh Kotecha, Abraham Korman, Huan Luo: manuscript preparation.

### Conflict of interest statement

The authors declare that the research was conducted in the absence of any commercial or financial relationships that could be construed as a potential conflict of interest.
